# Antibody glycosylation correlates with disease progression in SIV‐ *Mycobacterium tuberculosis* coinfected cynomolgus macaques

**DOI:** 10.1002/cti2.1474

**Published:** 2023-11-20

**Authors:** Ebene R Haycroft, Timon Damelang, Ester Lopez, Mark A Rodgers, Bruce D Wines, Mark Hogarth, Cassaundra L Ameel, Stephen J Kent, Charles A Scanga, Shelby L O'Connor, Amy W Chung

**Affiliations:** ^1^ Department of Microbiology and Immunology Doherty Institute for Infection and Immunity The University of Melbourne Melbourne VIC Australia; ^2^ Department of Microbiology and Molecular Genetics University of Pittsburgh School of Medicine Pittsburgh PA USA; ^3^ Immune Therapies Group Burnet Institute Melbourne VIC Australia; ^4^ Department of Clinical Pathology University of Melbourne Melbourne VIC Australia; ^5^ Department of Immunology and Pathology Monash University Melbourne VIC Australia; ^6^ Melbourne Sexual Health Centre and Department of Infectious Diseases, Alfred Hospital and Central Clinical School Monash University Melbourne VIC Australia; ^7^ Center for Vaccine Research University of Pittsburgh School of Medicine Pittsburgh PA USA; ^8^ Department of Pathology and Laboratory Medicine University of Wisconsin‐Madison Madison WI USA; ^9^ Wisconsin National Primate Research Centre University of Wisconsin‐Madison Madison WI USA

**Keywords:** antibodies, cynomolgus macaques, Fc, glycosylation, SIV, tuberculosis

## Abstract

**Objectives:**

Tuberculosis (TB) remains a substantial cause of morbidity and mortality among people living with human immunodeficiency virus (HIV) worldwide. However, the immunological mechanisms associated with the enhanced susceptibility among HIV‐positive individuals remain largely unknown.

**Methods:**

Here, we used a simian immunodeficiency virus (SIV)/TB‐coinfection Mauritian cynomolgus macaque (MCM) model to examine humoral responses from the plasma of SIV‐negative (*n* = 8) and SIV‐positive (*n* = 7) MCM 8‐week postinfection with *Mycobacterium tuberculosis* (*Mtb*).

**Results:**

Antibody responses to *Mtb* were impaired during SIV coinfection. Elevated inflammatory bulk IgG antibody glycosylation patterns were observed in coinfected macaques early at 8‐week post‐*Mtb* infection, including increased agalactosylation (G0) and reduced di‐galactosylation (G2), which correlated with endpoint *Mtb* bacterial burden and gross pathology scores, as well as the time‐to‐necropsy.

**Conclusion:**

These studies suggest that humoral immunity may contribute to control of TB disease and support growing literature that highlights antibody Fc glycosylation as a biomarker of TB disease progression.

## Graphical abstract

Diverging bulk IgG *N*‐linked glycan profiles are observed 8 weeks following *Mycobacterium tuberculosis* (Mtb) infection in simian immunodeficiency virus (SIV)‐naïve and SIV‐coinfected macaques.

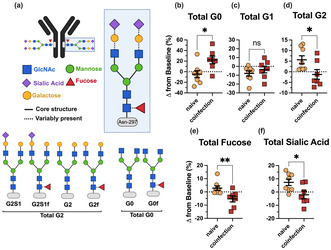

## Introduction

Tuberculosis (TB) remains a devastating global public health problem. In addition, circumstances of coinfection with *Mycobacterium tuberculosis* (*Mtb*) and human immunodeficiency virus (HIV) are a concerning issue.^1^, ^2^ Elevated risk of active tuberculosis (ATB), either from primary disease or reactivation from latent tuberculosis infection (LTBI), occurs early in HIV infection,^3^ with the risk of ATB being four‐to‐sevenfold higher among HIV‐positive persons than HIV‐naïve counterparts.^4^

Growing evidence in humans suggests that antibody immunity varies across the spectrum of TB disease states.^5^ Antibodies consist of Fab fragments that dictate antigen specificity, and a Fc region, that determine isotype or subclass. Prior studies in human cohorts suggest antibody responses to *Mtb* antigens are impaired by HIV coinfection, characterised by reduced titres and altered subclass profiles.^6^, ^7^ The Fc region coordinates various Fc‐effector functions, including engaging Fc‐receptors (FcRs), often found on innate immune cells. Antibodies from individuals with LTBI show elevated capacity to engage FcγRIIIa, linked with higher antibody‐dependent cellular cytotoxicity (ADCC), compared with individuals with ATB.^8^

The antibody Fc region is decorated with *N*‐linked glycans (or sugars). Differential patterns of antibody *N*‐linked glycans have been implicated across TB disease states and HIV infection and are potential biomarkers for disease progression.^5^, ^8^, ^9^, ^10^ Glycan profiles of bulk IgG antibodies from individuals with LTBI show higher levels of di‐galactose (two‐galactose sugars; G2) and sialic acid (S). Contrastingly, antibodies from individuals with ATB display higher proportions of agalactosylated (no‐galactose; G0) structures.^8^ Increased *N*‐linked agalactosylated and altered fucosylated structures are also observed in HIV infection.^11^ Nevertheless, the dual impact of HIV/*Mtb* coinfection on the *N*‐linked glycan antibody profile remains unknown.

Animal models for representing the spectrum of TB disease are limited.^12^ Chinese cynomolgus macaques (*Macaca fascicularis*) can exhibit the same spectrum of disease states following *Mtb* monoinfection—from LTBI to ATB—as observed in human infection.^13^, ^14^ Closely related Mauritian cynomolgus macaques (MCM) also display varied resistance to *Mtb* with a greater tendency towards ATB.^15^ MCM that develop ATB display hallmark clinical features of progressive TB disease, including human‐like granuloma formation and lung pathology.^15^, ^16^ Simian immunodeficiency virus (SIV)‐infected MCM also recapitulate the increased vulnerability to primary TB disease observed in HIV‐infected humans.^16^ While studies have shown dysregulated classical and nonclassical T cells in MCM coinfected with SIV/*Mtb*,^17^, ^18^, ^19^ the humoral compartment in this model remains uncharacterised.

Here, we examine the humoral profile following *Mtb* infection in SIV‐naïve and SIV‐coinfected MCMs. Using systems serology, we observed lower levels of *Mtb*‐specific antibodies in SIV‐coinfected animals than in SIV‐naïve animals, accompanied by lower engagement to macaque FcγRIIa and FcγRIIIa, suggesting reduced capacity to induce Fc‐effector functions. Additionally, distinct bulk IgG *N*‐linked sugar profiles were observed in SIV‐coinfected animals, characterised by increased agalactosylation. Interestingly, *N*‐linked sugar profiles at 8‐week post‐*Mtb* infection correlated with bacterial burden, gross pathology score and time‐to‐necropsy, regardless of SIV coinfection state, suggesting that they are a potential biomarker of TB disease progression during coinfection.

## Results

### SIV coinfection impairs *Mtb*‐specific humoral immunity

Plasma samples from (1) SIV‐naïve MCMs with *Mtb* infection (*n* = 8) and (2) SIV‐infected MCMs with *Mtb* coinfection (*n* = 7) were collected at two time points: baseline (prior to SIV or *Mtb* infection) and 8‐week post‐*Mtb* infection from a previously reported study by Rodgers *et al*.^16^ Anti‐SIV gp120 responses were observed 8‐week postinfection in SIV‐coinfected animals only (Supplementary figure 1). SIV‐naïve animals displayed variable disease outcomes (time‐to‐necropsy, median 16 weeks; IQR 11.75–20.25) and pathological manifestations, reflecting the spectrum of TB disease observed in humans (Table 1).^16^, ^20^ Contrastingly, all SIV/*Mtb*‐coinfected animals reached humane endpoint by 12 weeks following *Mtb* infection (time‐to‐necropsy, median 11 weeks; IQR 10–12),^16^ consistent with enhanced vulnerability to developing primary ATB disease (Table 1).^16^

**Table 1:** Characteristics of the Mauritian cynomolgus macaque (*Macaca fascicularis*) study cohort following *Mycobacterium tuberculosis* infection (adapted from Rodgers *et al.* ^16^)

	SIV‐naïve (*n* = 8)	SIV‐coinfected (*n* = 7)
Time‐to‐necropsy, weeks, median (IQR)	16 (11.75–20.25)	11 (10–12)
Total colony‐forming units (CFU), median (IQR)	4.71 × 10^5^ (1.92 × 10^5^ – 9.04 × 10^5^)	1.33 × 10^6^ (7.81 × 10^5^ – 8.01 × 10^6^)
Gross pathology score, median (IQR)	51 (44–71.75)	75 (59–85)

SIV, simian immunodeficiency virus.

**Figure 1:**
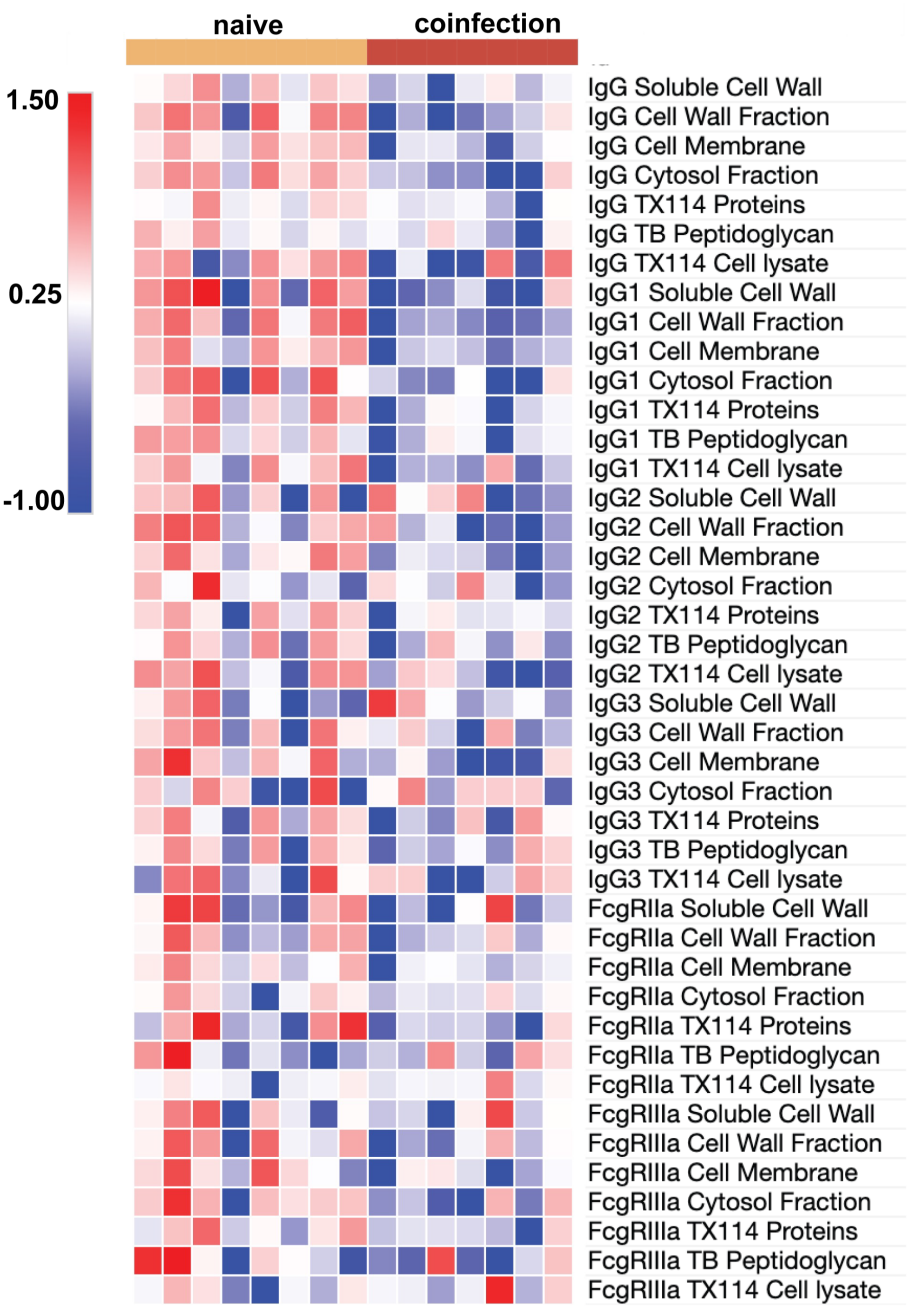
Simian immunodeficiency virus (SIV) infection impairs humoral immunity. Change from baseline (prior to any infection) in plasma humoral profiles (IgG, IgG1, IgG2, IgG3, FcγRIIa and FcγRIIIa) 8 weeks following infection with *Mycobacterium tuberculosis* (*Mtb*) in MCM without SIV (naïve; *n* = 8; yellow left bar) or coinfected with SIV (coinfection; *n* = 7; red right bar) as determined by multiplexing to *Mtb* soluble cell wall, cell wall fraction, cell membrane, cytosol fraction, TX114 proteins, peptidoglycan and cell lysate. Change in median fluorescence intensity data was normalised *via z*‐scoring before being represented as a heatmap (red indicates higher levels and blue indicates lower levels). Multiplex assays were repeated in duplicate.

Broad deficiencies in antibody responses elicited across a breadth of *Mtb* antigens were observed in SIV‐coinfected animals compared with SIV‐naïve animals at 8 weeks following *Mtb* infection (Figure 1). Increased IgG levels against fractions of *Mtb* cell membrane, cell wall, cytosol and soluble cell wall were observed in SIV‐naïve animals (all *P* = 0.01, respectively), while SIV‐coinfected animals had antibody levels comparable to baseline (Supplementary figure 1). Similar trends were recapitulated in subclass composition (IgG1, IgG2 and IgG3) with significant increases to *Mtb* cell membrane in SIV‐naïve animals (*P* = 0.009, 0.01, 0.03, respectively; Figure 1; Supplementary figure 2).

Anti‐*Mtb* antibodies that engage with FcγRIIIa are associated with greater ADCC activity and may be linked to more effective control of *Mtb*.^8^ Using soluble macaque FcγR‐dimer constructs shown to correlate with cell‐based *in vitro* Fc‐functional assays,^21^, ^22^, ^23^, ^24^ we observed impaired FcγR‐binding responses within SIV‐coinfected animals. FcγRIIIa‐dimerisation to *Mtb* cell membrane was significantly elevated in SIV‐naïve animals (*P* = 0.02). Contrastingly, responses in SIV‐coinfected animals remained comparable to baseline (Supplementary figure 2). Likewise, FcγRIIa‐binding—a surrogate measure of antibody‐dependent cellular phagocytosis (ADCP)—to fractions of *Mtb* cell wall and cell membrane trended higher in SIV‐naïve animals (*P* = 0.08 and 0.05, respectively), while FcγRIIa‐binding in SIV‐coinfected animals again remained comparable to baseline (Supplementary figure 2).

Taken together, our data provide evidence that SIV infection impairs the antibody response to *Mtb* early in infection, impairing the ability to engage potentially protective Fc‐effector functions.

### Altered IgG *N*‐linked sugar profiles in SIV coinfection

**Figure 2:**
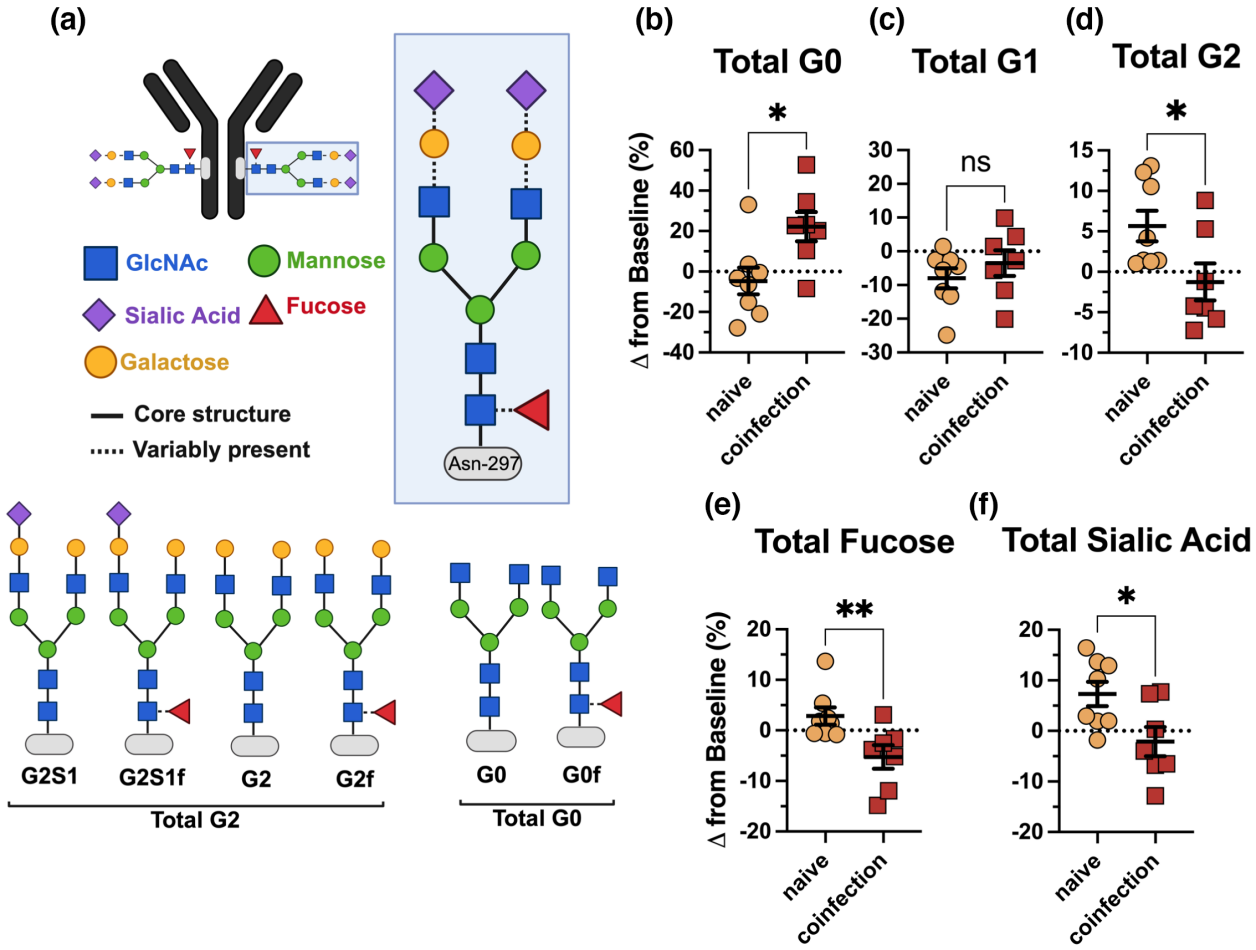
Diverging bulk IgG *N*‐linked glycan profile 8 weeks following *Mycobacterium tuberculosis* (*Mtb*) infection in simian immunodeficiency virus (SIV)‐naïve and SIV‐coinfected animals. **(a)** Schematic of a general *N*‐linked glycan structure, containing a *N*‐acetylglucosamine (GlcNac; blue square) and mannose (green circle) sugar core attached to asparagine (Asn)‐297 on the antibody heavy chain, with variable presence of galactose (G) (yellow circle), sialic acid (S) (purple diamond) and fucose **(f)** (red triangle) sugars attached, indicated by dotted lines. Examples of individual glycan structures are shown, reflecting the total glycan profile (Total G2 = G2S1, G2S1f, G2, G2f; Total G0 = G0, G0f). The percentage change in relative abundance from baseline (prior to any infection) of **(b)** total G0 (agalactosylated), **(c)** total G1 (mono‐galactosylated), **(d)** total G2 (di‐galactosylated), **(e)** total fucose and **(f)** total sialic acid glycans in bulk IgG as measured *via* capillary gel electrophoresis, 8 weeks following Mtb infection in MCM SIV‐naïve (naïve; *n* = 8; yellow circles) or coinfected with SIV (coinfection; *n* = 7; red squares). Glycosylation was determined using LabChip GXII Microchip‐CE electrophoresis. The Mann–Whitney *U*‐test was used to assess significance. *P*‐values: * < 0.05; **< 0.01; *** < 0.001; **** < 0.0001.

In addition to subclass and titre, variation can be further introduced to antibodies *via* the post‐translational addition of *N*‐linked glycans (or sugars) in the Fc region (Figure 2a).^25^ These sugars include a core containing *N*‐acetylglucosamine and mannose sugars, with variable presence of galactose (G), sialic acid (S) and fucose (f) sugars, giving rise to different glycan structures (Figure 2a).

Increases in *total* agalactosylated (zero galactose sugars; G0) glycan structures were observed in SIV‐coinfected animals compared with SIV‐naïve (*P* = 0.02; Figure 2b). Increases in G0 are often considered ‘inflammatory’ and have been associated with ATB.^8^ The proportion of *total* mono‐galactosylated (one galactose sugar; G1) glycan structures was similar in both SIV‐naïve and SIV‐positive MCM after *Mtb* infection (*P* = 0.4; Figure 2c; Supplementary figure 3). However, levels of *total* di‐galactosylated (two‐galactose sugars; G2) were significantly higher in SIV‐naïve animals than in SIV‐coinfected (*P* = 0.04; Figure 2d; Supplementary figure 3). Higher levels of G2 previously have been described in human LTBI cohorts,^8^ and higher G2 signatures may reflect the subset of SIV‐naïve animals that displayed less severe TB disease outcome (Table 1).

Given that galactose species such as G2 are precursor for sialylation (single‐S, S1; two‐S, S2), we measured S‐residues and found lower levels of *total*‐S species in SIV‐coinfected animals than in SIV‐naïve (*P* = 0.02; Figure 2a and f). Lower levels of *total* fucose also were observed in SIV‐coinfected animals (*P* = 0.009; Figure 2e; Supplementary figure 3). These collectively aligned with the increase in G2f and G2S1f glycan species in SIV‐naïve animals and their corresponding decrease in SIV‐coinfected animals (*P* = 0.009 and 0.003, respectively). Similar glycosylation patterns have been reported in antibodies from humans with LTBI.^8^

Collectively, these data suggest that, following 8 weeks of *Mtb* infection, SIV‐coinfected animals have altered global glycan structures relative to SIV‐naïve animals. The most notable shifts are enriched agalactosylated and reduced fucose and sialic acid structures in the SIV‐coinfected animals.

### Disease progression correlates with total IgG *N*‐linked glycan profile

Shifts in *N*‐linked glycan profile across different disease states, including TB, have been postulated to serve as a potential predictive biomarker of disease progression, with their changes often linked with immune activation. As previously reported, lower total *Mtb* bacterial burdens, measured in colony‐forming units (CFU), were observed in SIV‐naïve animals (median 4.71 × 10^5^ CFU; IQR 1.92 × 10^5^–9.04 × 10^5^ CFU) compared with SIV‐coinfected animals (median 1.33 × 10^6^ CFU; IQR 7.81 × 10^5^–8.01 × 10^6^ CFU).^16^ While this difference did not quite reach significance (*P* = 0.09), it likely reflects the less severe TB disease outcomes observed in SIV‐naïve group (Table 1).

**Figure 3:**
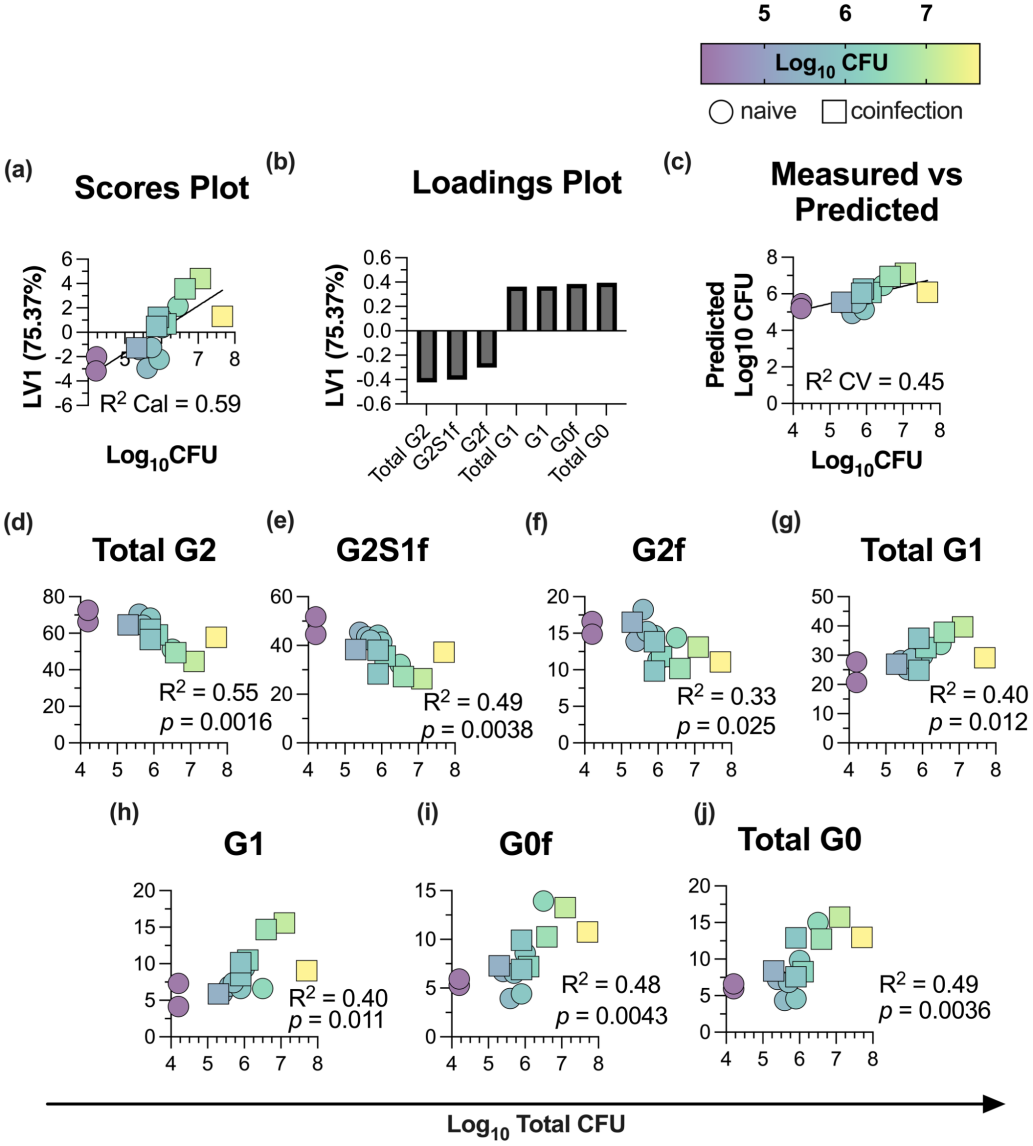
Bulk IgG *N*‐linked glycan profiles 8 weeks following *Mycobacterium tuberculosis* (*Mtb*) infection correlate with bacterial burden. Feature selection (elasticNet) identified seven *N*‐linked glycan features measured from bulk IgG of MCM (*n* = 15) 8 weeks following *Mtb* infection that were most strongly associated with bacterial loads, measured by log_10_ colony CFU. Partial least squares regression (PLS‐R) analysis condensed the variance of the seven‐feature selected *N*‐linked glycan antibody features into one latent variable (LV1), with 75.37% of the variance captured across LV1. The PLS‐R analysis demonstrated a *R* ^2^ calibration = 0.59 and *R* ^2^ cross‐validation = 0.45. The Score plot shows all **(a)** LV‐1 scores calculated for each animal plotted against respective log_10_ total CFU values. Simian immunodeficiency virus (SIV)‐naïve animals (*n* = 8) are indicated by circles and SIV‐coinfected animals (*n* = 7) by squares and further coloured over a spectrum representing log_10_ total CFU values (low score, dark purple; high score, yellow). **(b)** Loadings plot for 7 *N*‐linked glycan features represent the contribution of each feature to the score plot. Plot of **(c)** actual log_10_ total CFU (measured; *x*‐axis) against the log_10_ total CFU predicted by the PLS‐R analysis (predicted; *y*‐axis). Linear correlation of the relative abundance of **(d)** total G2 (di‐galactosylated glycans), **(e)** G2S1f **(f)** G2f, **(g)** Total G1 (mono‐galactosylated glycans), **(h)** G1, **(i)** G0f and **(j)** Total G0 (agalactosylated glycans) 8 weeks after *Mtb* infection with log_10_ total CFU values of each animal.

Thus, we used systems serology to determine whether glycan signatures measured at 8‐week post‐*Mtb* infection correlate with individual total bacterial burdens of the seven SIV‐positive and eight SIV‐naïve animals. Feature selection (elasticNet) identified seven glycan signatures most strongly associated with total CFU values. Next, partial least squares regression (PLS‐R) analysis was used to condense the variance found across these seven glycan signatures into one latent variable (LV1) for each animal. Using the LV1 scores obtained for each animal, we observed a moderate‐to‐strong relationship to respective log_10_ total CFU values (*R* ^2^ calibration = 0.59; dark colour, lower total log_10_ CFU; yellow, higher total log_10_ CFU; Figure 3a). The contribution of each of the seven glycan features driving this distribution on the score plot is directionally depicted in the corresponding loading plot (Figure 3b). Glycans such as G0 and G1 were associated with higher total CFU values (positive values on *y*‐axis), whereas G2‐based signatures were linked with lower total CFU values (negative values on *y*‐axis). These analyses suggest that distinct glycan profiles are correlative markers of TB disease severity. Collectively, the glycan profiles were moderately associated with total bacterial burdens of study animals (*R* ^2^ cross‐validation = 0.45; Figure 3c).

To confirm this association between glycans and TB disease severity, we individually correlated each glycan with log_10_ total bacterial load for each animal. *Total*‐G2, G2S1f and G2f displayed moderate inverse correlations with log_10_ total CFU (*R* ^2^ = 0.55, *P* = 0.0016; *R* ^2^ = 0.49, *P* = 0.0038; *R* ^2^ = 0.33, *P* = 0.025, respectively; Figure 3d–f). Conversely, *Total*‐G1, G1, G0f and *Total*‐G0 showed a moderate positive correlation with log_10_ total CFU (*R* ^2^ = 0.40, *P* = 0.012; *R* ^2^ = 0.40, *P* = 0.01; *R* ^2^ = 0.48, *P* = 0.0043; *R* ^2^ = 0.49, *P* = 0.0036, respectively; Figure 3g–j). As before, distinct trends in glycan abundance were distributed across different total CFU values.

Given this, we next assessed the relationship of glycans to other clinical parameters previously reported for the 15 MCM (seven SIV‐positive, eight SIV‐naïve) after *Mtb* infection.^16^ Like total CFU, similar trends were observed when *N*‐linked glycans were correlated with time‐to‐necropsy and gross pathology scores, indicating these associations with glycans are robust over multiple clinical parameters (Supplementary figures 4–6). Interestingly, given that these sugar signatures were captured at a relatively early time point after *Mtb* infection (8 weeks), this highlights the potential for antibody glycosylation to serve as discriminatory biomarkers of disease progression in *Mtb* infection, irrespective of SIV status.

## Discussion

We used a previously described cohort of MCMs^16^ to profile the humoral responses elicited 8 weeks after *Mtb* infection of SIV‐naïve and SIV‐positive animals. A key finding was the change in bulk IgG glycan profile of SIV‐coinfected animals—largely characterised by increased agalactosylation, reduced fucosylation and reduced sialyation—compared with SIV‐naïve animals following *Mtb* infection. Changes in bulk IgG glycosylation profiles have also been observed in other acute and chronic infectious diseases and have been well described to reflect the increased inflammatory state and severity of individuals with a range of autoimmune diseases.^5^, ^26^, ^27^, ^28^ Reduced fucosylation has been described previously in humans following HIV infection.^11^ Likewise, changes in bulk IgG glycan profiles towards agalactosylation are observed early during acute (first 5 months) HIV infection and in ATB disease in human cohorts.^8^, ^9^, ^11^ It remains unclear in our study whether the decreased levels of galactose are the consequence of SIV infection or exacerbated by *Mtb* coinfection. Agalactosylated bulk IgG antibodies are associated with more inflammatory disease profiles, including HIV, COVID‐19 and TB, as well as in patients with autoimmune diseases.^5^, ^8^, ^9^ In this study, agalactosylated antibodies moderately correlated with total bacterial burden, time‐to‐necropsy and gross pathology, further supporting the potential for antibody glycosylation to serve as a biomarker for TB disease progression. Interestingly, analysis of glycan profiles as a whole was more correlative than examining singular glycan structures as multivariate analysis can capture global changes in glycosylation, likely given there are several potential glycan structures that can be associated with inflammatory or anti‐inflammatory states.^5^, ^26^

In addition to changes in bulk IgG glycan structures, reduced levels of *Mtb*‐specific antibodies and FcγR‐binding were apparent 8 weeks following *Mtb* infection in SIV‐coinfected animals. Human studies have provided evidence for antibody titres against specific *Mtb* antigens. Indeed, in the MVA85A TB vaccine trial, IgG response against Ag85 was identified *ad hoc* to be associated with protection from TB disease in infants.^29^ Furthermore, recent work from studies in high TB burden settings showed that higher *Mtb*‐specific IgG3 titres correlated with protection from recurrent *Mtb* infections.^30^ Our data with pooled antigens indicate a wide deficiency in the breadth of antibody response, likely including these compromised antigen‐specific responses. Our findings suggest impaired serum humoral responses against *Mtb* occur relatively early (< 6 months) following SIV infection. The SIV‐coinfected animals studied here had lower total CD4^+^ T‐cell counts at the time of *Mtb* coinfection,^16^ and it is possible that the reduced T‐cell repertoire may impair the antibody response given the important roles of CD4^+^ T cells in providing B‐cell help.^31^

Our work was performed with a small cohort of 15 macaques (seven SIV‐positive, eight SIV‐naïve), which may limit the strength of our conclusions. Whether these observations apply to human cohorts remains to be confirmed. A further limitation of our study is that we did not assess the antigen‐specific antibodies for their glycosylation profiles or confirm deficiencies in *Mtb*‐specific FcR‐binding with cell‐based assays. In our study, low *Mtb* antigen‐specific antibody titres were observed. Importantly, our samples were taken early in infection; and this may reflect the slow‐growing nature of TB, compounded by a low‐dose bronchoscopic infection model, notably with prior work demonstrating a link between bacterial burden and antibody titres.^5^ Thus, with limited plasma sample volumes, and low *Mtb*‐specific antibody titres, antigen‐specific IgG antibodies were not extracted and examined. However, future work will include larger animal cohorts, where we may also be able to examine the influence of SIV disease severity upon Mtb disease progression and further test whether antigen‐specific glycosylation patterns represent a robust biomarker for TB disease progression.

In conclusion, our study reveals a diverging humoral landscape to *Mtb* in SIV‐coinfected animals compared with SIV‐naïve animals 8 weeks following *Mtb* infection. Our data also suggest that alterations in the global glycosylation profile of antibodies may serve as a biomarker for TB disease severity, regardless of SIV status.

## Methods

### Animal samples and ethics statement

The Mauritian cynomolgus macaques (*M. fascicularis*; MCM) plasma samples used were provided from a previously reported study by Rodgers *et al*.^16^ which analysed the susceptibility to *Mtb* infection in MCM with pre‐existing SIV infection (Table 1). The care of these animals and plasma collection procedures have been described extensively.^16^ All animal work was approved by the Institutional Animal Care and Use Committee at the University of Pittsburgh and was in accordance with the Animal Welfare Act and *Guide for the Care and Use of Laboratory Animals* (8th edition).^16^

Plasma samples were collected before any SIV or *Mtb* infection (baseline samples). Briefly, seven adult (> 4 years of age) MCM were initially infected intrarectally to SIV_mac239_ (3000 TCID_50_). Following 6 months, all animals were infected with a low dose (3–12 CFU) of *Mtb* Erdman strain using a bronchoscope. Another eight SIV‐negative adult MCM were identically infected only with *Mtb*. Plasma samples were collected at 8‐week post‐SIV and/or *Mtb* infection.

### Antigens

*Mtb* antigens of the H37Rv strain (sourced from BEI resources) are as follows: soluble cell wall proteins (NR‐14840), cell wall fraction (NR‐14828), cell membrane proteins (NR‐14831), cytosol fraction (NR‐14834), purified peptidoglycan (NR‐14853) and TX‐114 soluble proteins (NR‐14831). Additionally, a SIV_mac251_ gp120 antigen (Sino Biological, Beijing, China; 40410‐V08H‐100) was used as a control.

### *Mtb*‐specific multiplex array

A custom multiplex array was developed to analyse *Mtb* antigen‐specific antibodies in MCM plasma samples pre‐ and postinfection. Antigens listed above were initially covalently coupled onto carboxylated magnetic beads (Bio‐Rad, Hercules, CA, USA) using established protocols as previously described.^8^, ^23^ The antigen‐coated beads (1000 of each per well) were added to black, clear‐bottom 96‐well microplates (Greiner Bio‐One, Kremsmünster, Austria) followed by serum diluted 1:100 in assay buffer (0.01%BSA/PBS) and then incubated on a plate shaker overnight at 4°C. Plates were washed with PBS 0.05% Tween (PBST) using a Bio‐Plex Pro magnetic plate washer. A range of different detectors to profile features of the antigen‐specific antibodies were used. Mouse anti‐pan macaque antibodies that detect the isotypes IgG (Mabtech, Cincinnati, OH, USA; 3850‐6‐250) and IgA (Mabtech; 3860M‐1H‐6), the rhesus macaque subclasses IgG1, IgG2 and IgG3 (NIH NHP reagents), or biotinylated dimeric macaque FcγR,^32^ were added at 1.3 μg mL^−1^ (50 μL per well) and incubated on a plate shaker for 2 h at room temperature (RT). For all dimeric FcγR reagents, plates were rewashed and PE‐conjugated streptavidin was added to wells and incubated for an extra 1 h on a plate shaker at RT. For all isotypes and subclasses, a goat anti‐mouse IgG‐PE (Life Tech) secondary was used. Following incubation, plates were washed and resuspended in sheath fluid (ThermoFisher, Scoresby, Australia) in accordance with the manufacturer's instructions. A Magpix multiplex system running x‐PONENT software was used for acquisition. The signal reported is defined as the median fluorescence intensity (MFI) of the PE for each microsphere. The background was determined by the MFI of wells with microspheres and assay buffer only and subtracted from the MFI of each sample in this assay.

### IgG purification and quantification

Total IgG was purified from plasma using a Melon Gel IgG spin purification kit (ThermoFisher) following the manufacturer's instructions. In brief, Melon Gel resin was added to Pierce Spin Columns (ThermoFisher), followed by serum diluted 1:10 in Melon Gel purification buffer. Following centrifugation, eluted IgG was buffered‐exchanged into phosphate buffered saline (PBS) using 100 kDa Amicon Ultra centrifugal filters (Merck Millipore, Burlington, MA, USA). Purified IgG concentrations were determined by a human IgG enzyme‐linked immunosorbent assay (ELISA) kit (Mabtech) following the manufacturer's instructions.

### Measurement of IgG *N*‐glycans

The *N*‐linked glycan profile of purified IgG was measured using the ProfilerPro Glycan Profiling Kit (PerkinElmer, Waltham, MA, USA) following the manufacturer's instructions. In brief, equal quantities (1060 μg) of IgG were denatured prior to treatment with PNGase enzyme to cleave the *N*‐linked glycans. The released glycans were labelled and reconstituted in water before being read using capillary gel electrophoresis (LabChip GXII Touch HT protein characterisation system) that can measure the following glycan species: G0, G0f, G1, G1f, G2, G2f, G2S21, G2S1f. Analysis was performed using LabChip GX Reviewer 5.1 software. Total glycans were used to assess bulk changes (Total G0 = G0 + G0f; Total G1 = G1 + G1f; Total G2 = G2 + G2f + G2S21 + G2S1f; Total Fucose = G0f + G1f + G2f + G2S1f; Total Sialic Acid = G2S1 + G2S1f).

### Statistical analysis

Prism GraphPad version 8.0 (GraphPad Software, La Jolla, CA, USA) and MATLAB version 9.6 (The MathWorks, Inc., Natick, MA, USA) were used for statistical analysis. Groups were compared by either the Kruskal–Wallis test with Dunn's multiple comparisons, or the Mann–Whitney *U*‐test where appropriate. Pearson correlation was used to correlate individual glycan features with outcomes (e.g. gross pathology scores). A *P*‐value of 0.05 was set as the level for statistical significance. Values used in heat maps were first standardised by calculating respective *z*‐scores and generated using Morpheus (https://software.broadinstitute.org/morpheus).

### Multivariate analysis

All glycan data were first preprocessed by *z*‐scoring. Partial least squares regression (PLS‐R) was performed on glycan features to visualise the relationship between glycan signatures with continuous variables (i.e. log_10_ total CFU) of study animals using Eigenvector PLS toolbox (Eigenvector, Washington State, USA). Leave‐one‐out cross‐validation was used. Because of a sample size of *n* = 15, the data set was used to calibrate the PLS‐R analysis and was not further split to create a test set (i.e. validation set) for a predictive model.

## Author contributions

**Ebene R Haycroft:** Data curation; formal analysis; investigation; methodology; visualization; writing – original draft; writing – review and editing. **Timon Damelang:** Investigation; methodology; writing – review and editing. **Ester Lopez:** Investigation; methodology. **Mark A Rodgers:** Investigation. **Bruce D Wines:** Resources. **Mark Hogarth:** Resources. **Cassaundra L Ameel:** Investigation. **Stephen J Kent:** Funding acquisition; investigation; writing – review and editing. **Charles A Scanga:** Funding acquisition; investigation; project administration; writing – review and editing. **Shelby L O'Connor:** Conceptualization; data curation; funding acquisition; investigation; project administration; writing – original draft; writing – review and editing. **Amy W Chung:** Conceptualization; data curation; formal analysis; funding acquisition; investigation; methodology; project administration; supervision; writing – original draft; writing – review and editing.

## Conflict of interest

The authors declare no conflict of interest.

## Supporting information


Supplementary figures 1–6

## Data Availability

The data that support the findings of this study are available from the corresponding author upon reasonable request.

## References

[1] Bagcchi S . WHO's global tuberculosis report 2022. Lancet Microbe 2023; 4: e20. 36521512 10.1016/S2666-5247(22)00359-7

[2] Ford N , Shubber Z , Meintjes G *et al*. Causes of hospital admission among people living with HIV worldwide: a systematic review and meta‐analysis. Lancet HIV 2015; 2: e438–e444. 26423651 10.1016/S2352-3018(15)00137-X

[3] Sonnenberg P , Glynn JR , Fielding K , Murray J , Godfrey‐Faussett P , Shearer S . How soon after infection with HIV does the risk of tuberculosis start to increase? A retrospective cohort study in South African gold miners. J Infect Dis 2005; 191: 150–158. 15609223 10.1086/426827

[4] Gupta A , Wood R , Kaplan R , Bekker L‐G , Lawn SD . Tuberculosis incidence rates during 8 years of follow‐up of an antiretroviral treatment cohort in South Africa: comparison with rates in the community. PLoS One 2012; 7: e34156. 22479548 10.1371/journal.pone.0034156 PMC3316623

[5] McLean MR , Lu LL , Kent SJ , Chung AW . An inflammatory story: antibodies in tuberculosis comorbidities. Front Immunol 2019; 10: 2846. 31921122 10.3389/fimmu.2019.02846 PMC6913197

[6] van Woudenbergh E , Irvine EB , Davies L *et al*. HIV is associated with modified humoral immune responses in the setting of HIV/TB coinfection. mSphere 2020; 5: e00104‐20. 32434838 10.1128/mSphere.00104-20 PMC7380575

[7] Da Costa C , Khanolkar‐Young S , Elliott A , Wasunna K , McAdam K . Immunoglobulin G subclass responses to mycobacterial lipoarabinomannan in HIV‐infected and non‐infected patients with tuberculosis. Clin Exp Immunol 1993; 91: 25–29. 8419082 10.1111/j.1365-2249.1993.tb03348.x PMC1554641

[8] Lu LL , Chung AW , Rosebrock TR *et al*. A functional role for antibodies in tuberculosis. Cell 2016; 167: 433–443.e14. 27667685 10.1016/j.cell.2016.08.072 PMC5526202

[9] Lu LL , Das J , Grace PS , Fortune SM , Restrepo BI , Alter G . Antibody Fc glycosylation discriminates between latent and active tuberculosis. J Infect Dis 2020; 222: 2093–2102. 32060529 10.1093/infdis/jiz643 PMC7661770

[10] McLean MR , Wragg KM , Lopez E *et al*. Serological and cellular inflammatory signatures in end‐stage kidney disease and latent tuberculosis. Clin Transl Immunol 2021; 10: e1355. 10.1002/cti2.1355 PMC8569694 34765193

[11] Ackerman ME , Crispin M , Yu X *et al*. Natural variation in Fc glycosylation of HIV‐specific antibodies impacts antiviral activity. J Clin Invest 2013; 123: 2183–2192. 23563315 10.1172/JCI65708 PMC3637034

[12] Scanga CA , Flynn JL . Modeling tuberculosis in nonhuman primates. Cold Spring Harb Perspect Med 2014; 4: a018564. 25213189 10.1101/cshperspect.a018564 PMC4292094

[13] Capuano SV III , Croix DA , Pawar S *et al*. Experimental *Mycobacterium tuberculosis* infection of cynomolgus macaques closely resembles the various manifestations of human *M. tuberculosis* infection. Infect Immun 2003; 71: 5831–5844. 14500505 10.1128/IAI.71.10.5831-5844.2003 PMC201048

[14] Lin PL , Rodgers M , Smith LK *et al*. Quantitative comparison of active and latent tuberculosis in the cynomolgus macaque model. Infect Immun 2009; 77: 4631–4642. 19620341 10.1128/IAI.00592-09 PMC2747916

[15] Maiello P , DiFazio RM , Cadena AM *et al*. Rhesus macaques are more susceptible to progressive tuberculosis than cynomolgus macaques: a quantitative comparison. Infect Immun 2018; 86: e00505‐17. 10.1128/IAI.00505-17 PMC5778369 28947646

[16] Rodgers MA , Ameel C , Ellis‐Connell AL *et al*. Preexisting simian immunodeficiency virus infection increases susceptibility to tuberculosis in Mauritian cynomolgus macaques. Infect Immun 2018; 86: e00565‐18. 30224552 10.1128/IAI.00565-18 PMC6246917

[17] Ellis AL , Balgeman AJ , Larson EC *et al*. MAIT cells are functionally impaired in a Mauritian cynomolgus macaque model of SIV and Mtb co‐infection. PLoS Pathog 2020; 16: e1008585. 32433713 10.1371/journal.ppat.1008585 PMC7266356

[18] Larson EC , Ellis‐Connell A , Rodgers MA *et al*. Pre‐existing simian immunodeficiency virus infection increases expression of T cell markers associated with activation during early *Mycobacterium tuberculosis* coinfection and impairs TNF responses in granulomas. J Immunol 2021; 207: 175–188. 34145063 10.4049/jimmunol.2100073 PMC8683577

[19] Moriarty RV , Rodgers MA , Ellis AL *et al*. Spontaneous control of SIV replication does not prevent T cell dysregulation and bacterial dissemination in animals Co‐infected with *M. tuberculosis* . Microbiol Spectr 2022; 10: e0172421. 35467372 10.1128/spectrum.01724-21 PMC9241861

[20] Cadena AM , Fortune SM , Flynn JL . Heterogeneity in tuberculosis. Nat Rev Immunol 2017; 17: 691–702. 28736436 10.1038/nri.2017.69 PMC6247113

[21] Wines BD , Vanderven HA , Esparon SE , Kristensen AB , Kent SJ , Hogarth PM . Dimeric FcγR ectodomains as probes of the Fc receptor function of anti‐influenza virus IgG. J Immunol 2016; 197: 1507–1516. 27385782 10.4049/jimmunol.1502551

[22] McLean MR , Madhavi V , Wines BD , Hogarth PM , Chung AW , Kent SJ . Dimeric Fcγ receptor enzyme‐linked immunosorbent assay to study HIV‐specific antibodies: a new look into breadth of Fcγ receptor antibodies induced by the RV144 vaccine trial. J Immunol 2017; 199: 816–826. 28615419 10.4049/jimmunol.1602161

[23] Selva KJ , Van De Sandt CE , Lemke MM *et al*. Systems serology detects functionally distinct coronavirus antibody features in children and elderly. Nat Commun 2021; 12: 2037. 33795692 10.1038/s41467-021-22236-7 PMC8016934

[24] Haycroft ER , Davis SK , Ramanathan P *et al*. Antibody Fc‐binding profiles and ACE2 affinity to SARS‐CoV‐2 RBD variants. Med Microbiol Immunol 2023; 212: 291–305. 37477828 10.1007/s00430-023-00773-w PMC10372118

[25] Jefferis R , Lund J , Pound JD . IgG‐fc‐mediated effector functions: molecular definition of interaction sites for effector ligands and the role of glycosylation. Immunol Rev 1998; 163: 59–76. 9700502 10.1111/j.1600-065x.1998.tb01188.x

[26] Purcell RA , Theisen RM , Arnold KB , Chung AW , Selva KJ . Polyfunctional antibodies: a path towards precision vaccines for vulnerable populations. Front Immunol 2023; 14: 1183727. 37600816 10.3389/fimmu.2023.1183727 PMC10433199

[27] Seeling M , Brückner C , Nimmerjahn F . Differential antibody glycosylation in autoimmunity: sweet biomarker or modulator of disease activity? Nat Rev Rheumatol 2017; 13: 621–630. 28905852 10.1038/nrrheum.2017.146

[28] Zhou X , Motta F , Selmi C , Ridgway WM , Gershwin ME , Zhang W . Antibody glycosylation in autoimmune diseases. Autoimmun Rev 2021; 20: 102804. 33727152 10.1016/j.autrev.2021.102804 PMC8058319

[29] Fletcher HA , Snowden MA , Landry B *et al*. T‐cell activation is an immune correlate of risk in BCG vaccinated infants. Nat Commun 2016; 7: 11290. 27068708 10.1038/ncomms11290 PMC4832066

[30] Fischinger S , Cizmeci D , Shin S *et al*. A *Mycobacterium tuberculosis* specific IgG3 signature of recurrent tuberculosis. Front Immunol 2021; 12: 729186. 34630406 10.3389/fimmu.2021.729186 PMC8493041

[31] Carpenter SM , Lu LL . Leveraging antibody, B cell and Fc receptor interactions to understand heterogeneous immune responses in tuberculosis. Front Immunol 2022; 13: 830482. 35371092 10.3389/fimmu.2022.830482 PMC8968866

[32] Parsons MS , Lee WS , Kristensen AB *et al*. Fc‐dependent functions are redundant to efficacy of anti‐HIV antibody PGT121 in macaques. J Clin Invest 2019; 129: 182–191. 30475230 10.1172/JCI122466 PMC6307963

