# Supplementary material for: Antibody glycosylation correlates with disease progression in SIV‐ Mycobacterium tuberculosis coinfected cynomolgus macaques
Source: Clin Transl Immunology. 2023 Nov 20;12(11):e1474. doi: 10.1002/cti2.1474 (PMC10660403; doi:10.1002/cti2.1474)
Supplement: Supplementary file 1 — Supplementary figures 1–6 [file CTI2-12-e1474-s001.pdf]

Supporting Information

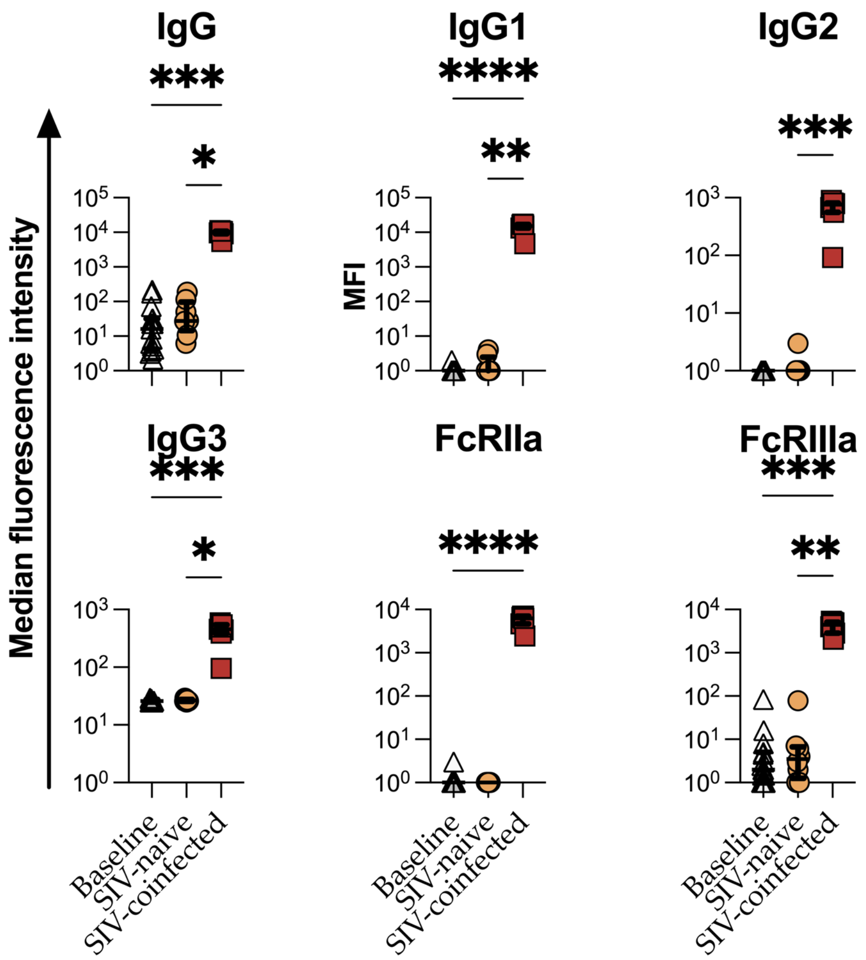

Supplementary Figure 1. Antibody IgG responses to SIVgp120 in the plasma of SIV-naïve and SIV-coinfected MCM as determined by multiplex assay. Antibody isotype (IgG), subclass (IgG1, IgG2, IgG3), and macaque FcR-binding (FcγRIIa, and FcγRIIIa) profiles to SIVgp120 of MCM at baseline (black; n = 15) and eight weeks following infection with *Mycobacterium tuberculosis* (*Mtb*) in SIV-naïve (yellow; n = 8) and SIV-coinfected (red; n = 7) as determined by multiplexing. The Kruskal-Wallis test with Dunn's multiple comparisons was used to assess statistical significance. *P*-values: * < 0.05; ** < 0.01; *** < 0.001; **** < 0.0001. Multiplex assays were repeated in duplicate.

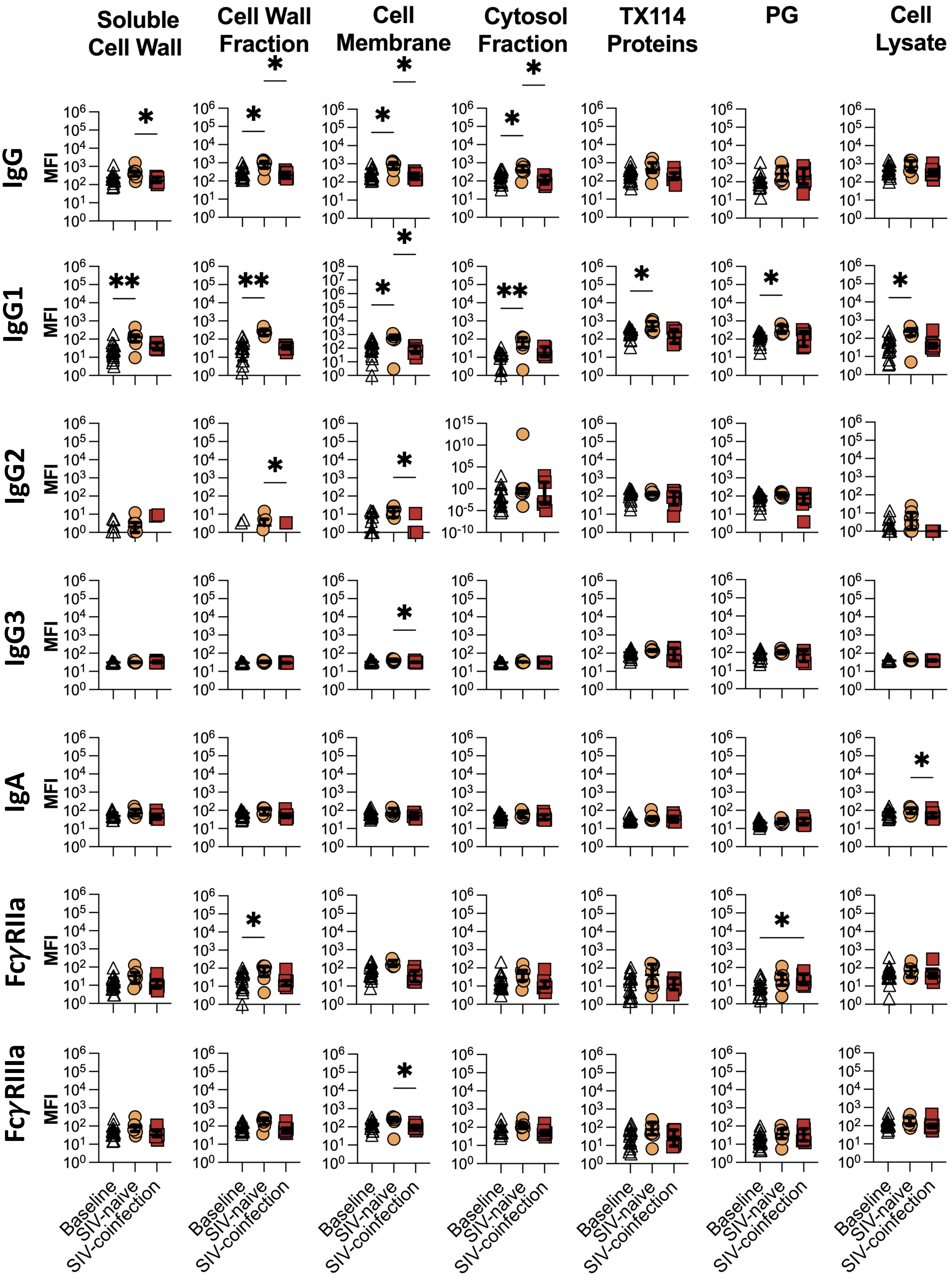

Supplementary Figure 2. (Cont. next page)

Supplementary Figure 2. Anti-*Mtb* humoral profiles of simian immunodeficiency virus (SIV)-naïve and SIV-coinfected MCM as determined by multiplex assay. Antibody isotype (IgG, IgA), subclass (IgG1, IgG2, IgG3) and FcγR-binding (FcγRIIa, FcγRIIIa) profiles of MCM at baseline (prior to any infection; black; *n* = 15) and eight weeks following infection with *Mycobacterium tuberculosis* (*Mtb*) in SIV-naïve (yellow; *n* = 8) and SIV-coinfected (red; *n* = 7) as determined by multiplexing to *Mtb* soluble cell wall, cell wall fraction, cell membrane, cytosol fraction, TX114 proteins, peptidoglycan (PG) and cell lysate. The Kruskal-Wallis test with Dunn's multiple comparisons was used to assess statistical significance. *P*-values: * < 0.05; ** < 0.01; *** < 0.001; **** < 0.0001. Multiplex assays were repeated in duplicate.

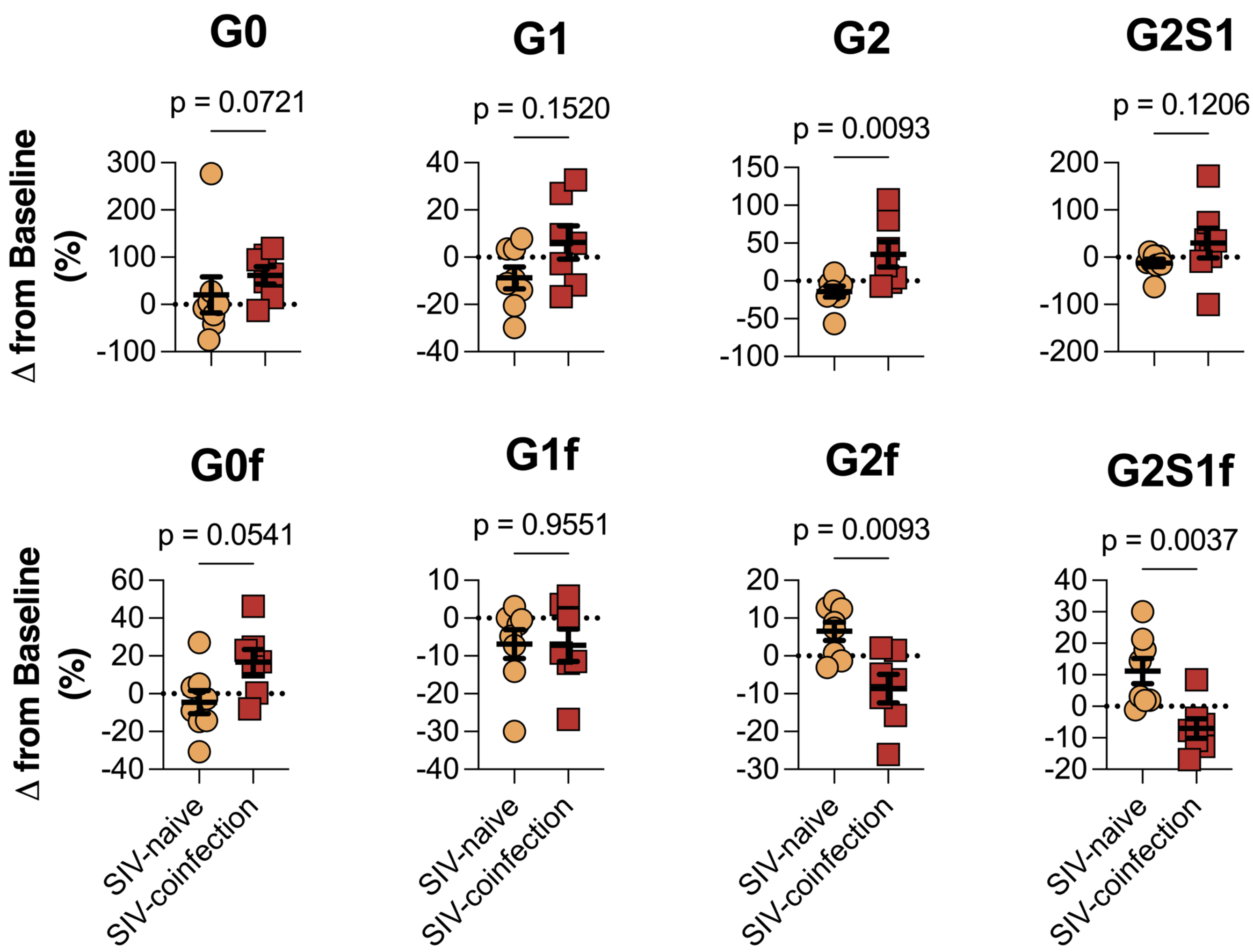

Supplementary Figure 3. Change in relative abundance of bulk IgG *N*-linked glycan following *Mtb* infection in SIV-naïve and SIV-coinfected macaques. The percentage change in relative abundance from baseline (prior to any infection) of individual glycans (G0, G0f, G1, G1f, G2, G2f, G2S1, G2S1f) eight weeks following infection with *Mtb* in MCM naïve for SIV (SIV-naïve; n = 8; yellow circles) and coinfected with SIV (SIV-coinfection; n = 7; red squares). The Mann-Whitney *U*-test used to assess significance. *P*-values: * < 0.05; ** < 0.01; *** < 0.001; **** < 0.0001.

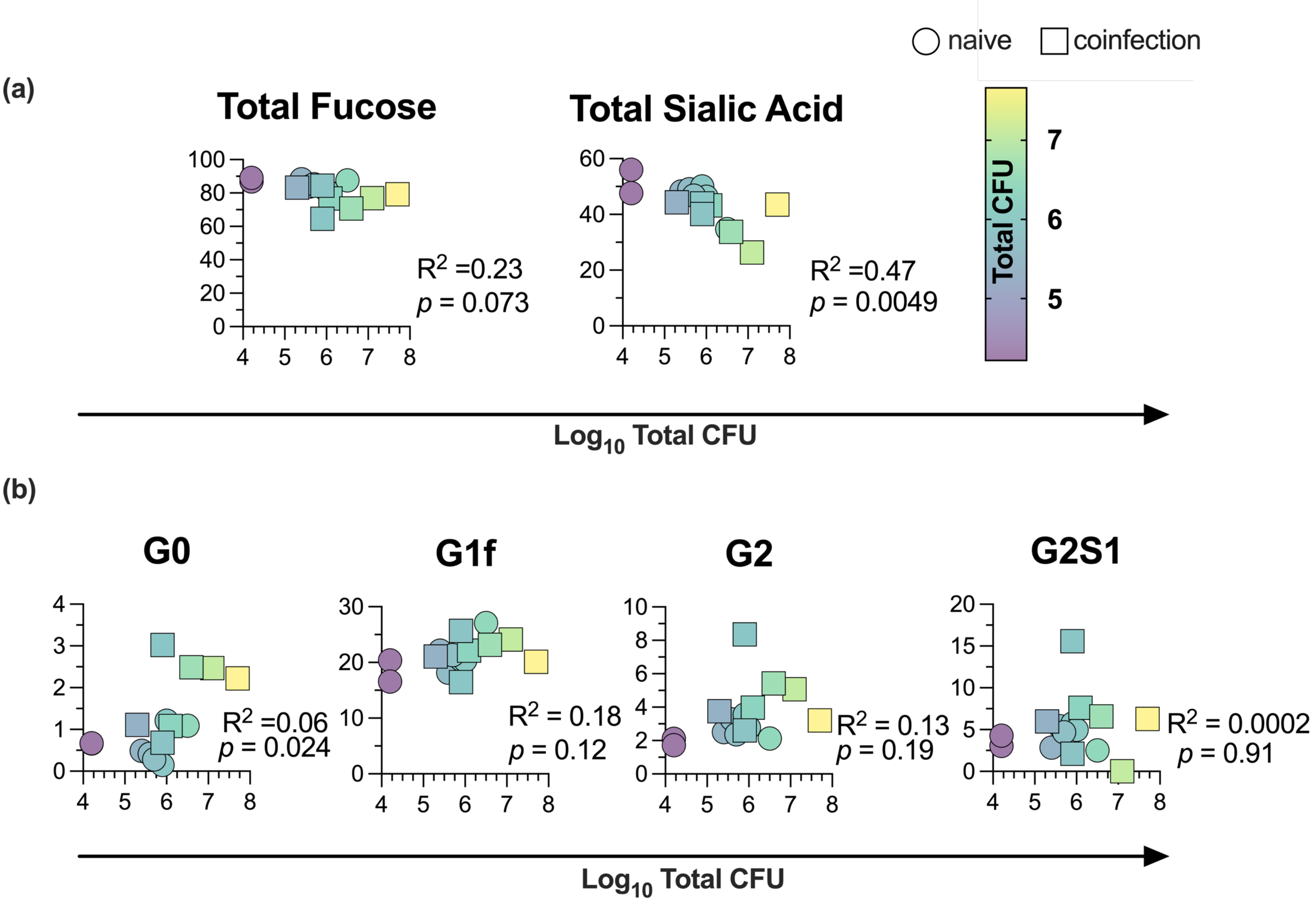

Supplementary Figure 4. Correlation of bacterial burden with total and individual *N*-linked glycans eight weeks after *Mtb* infection. Linear correlation of the relative abundance of **(a)** total fucose and total sialic acid and **(b)** individual glycans G0, G1f, G2, and G2S1 eight weeks following *Mtb* infection with bacterial burden, reported as $\log_{10}$ CFU, from each animal. Simian immunodeficiency virus (SIV)-naïve animals (n = 8) are indicated by circles and SIV-coinfected animals (n =7) by squares, further coloured over a spectrum representing $\log_{10}$ total CFU values (low score, dark purple; high score, yellow).

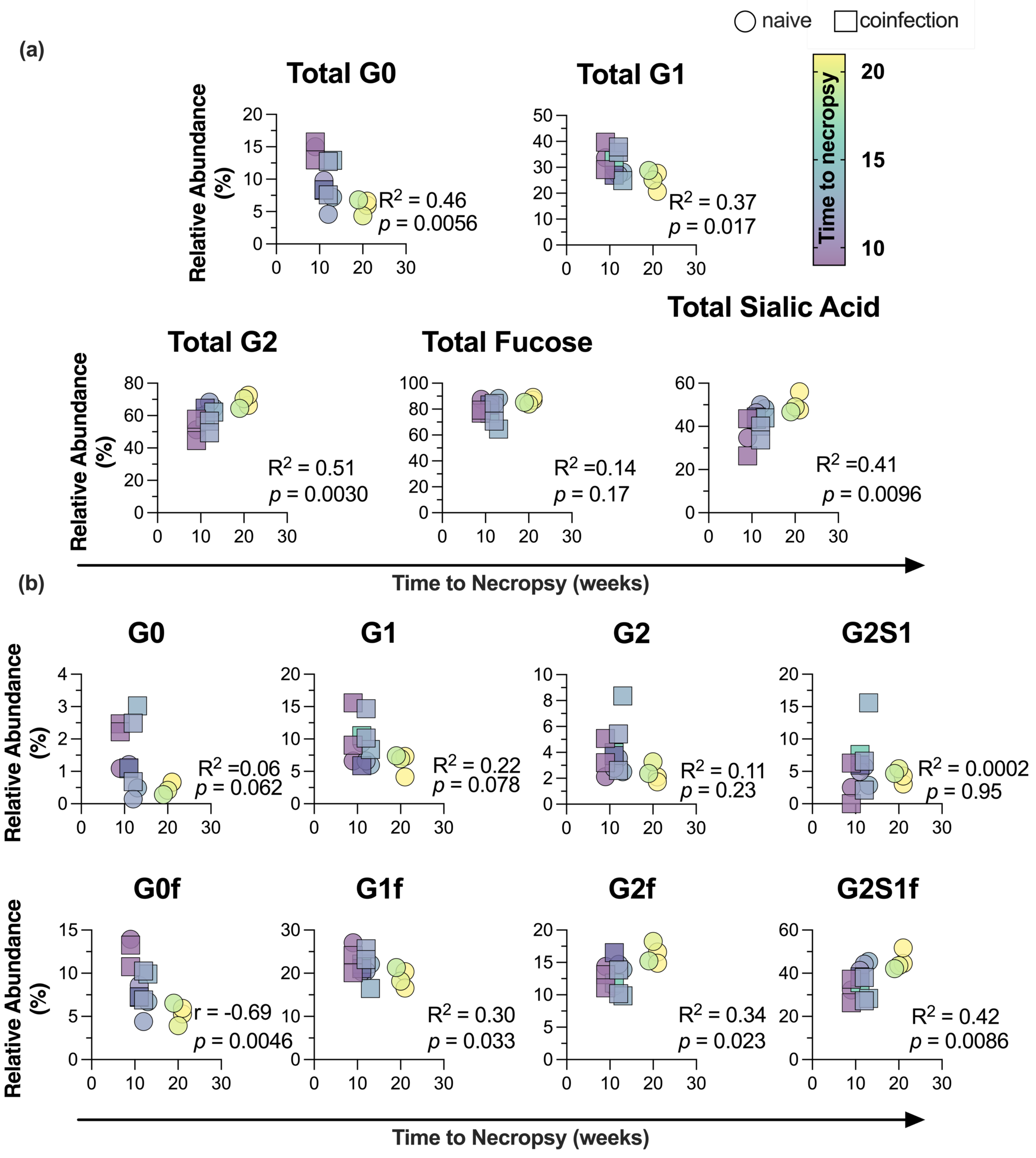

Supplementary Figure 5. Correlation of time-to-necropsy with total and individual *N*-linked glycans eight weeks following *Mtb* infection. Linear correlation of the relative abundance of **(a)** total G0**,** total G1, total G2, total fucose, and total sialic acid and **(b)** individual glycans G0, G0f, G1, G1f, G2, G2f, G2S1, G2S1f eight weeks following *Mtb* infection with time-to-necropsy for each animal. Simian immunodeficiency virus (SIV)-naïve animals (n = 8) are indicated by circles and SIV-coinfected animals (n =7) by squares, further coloured over a spectrum representing time to necropsy (weeks) values (low score, dark purple; high score, yellow).

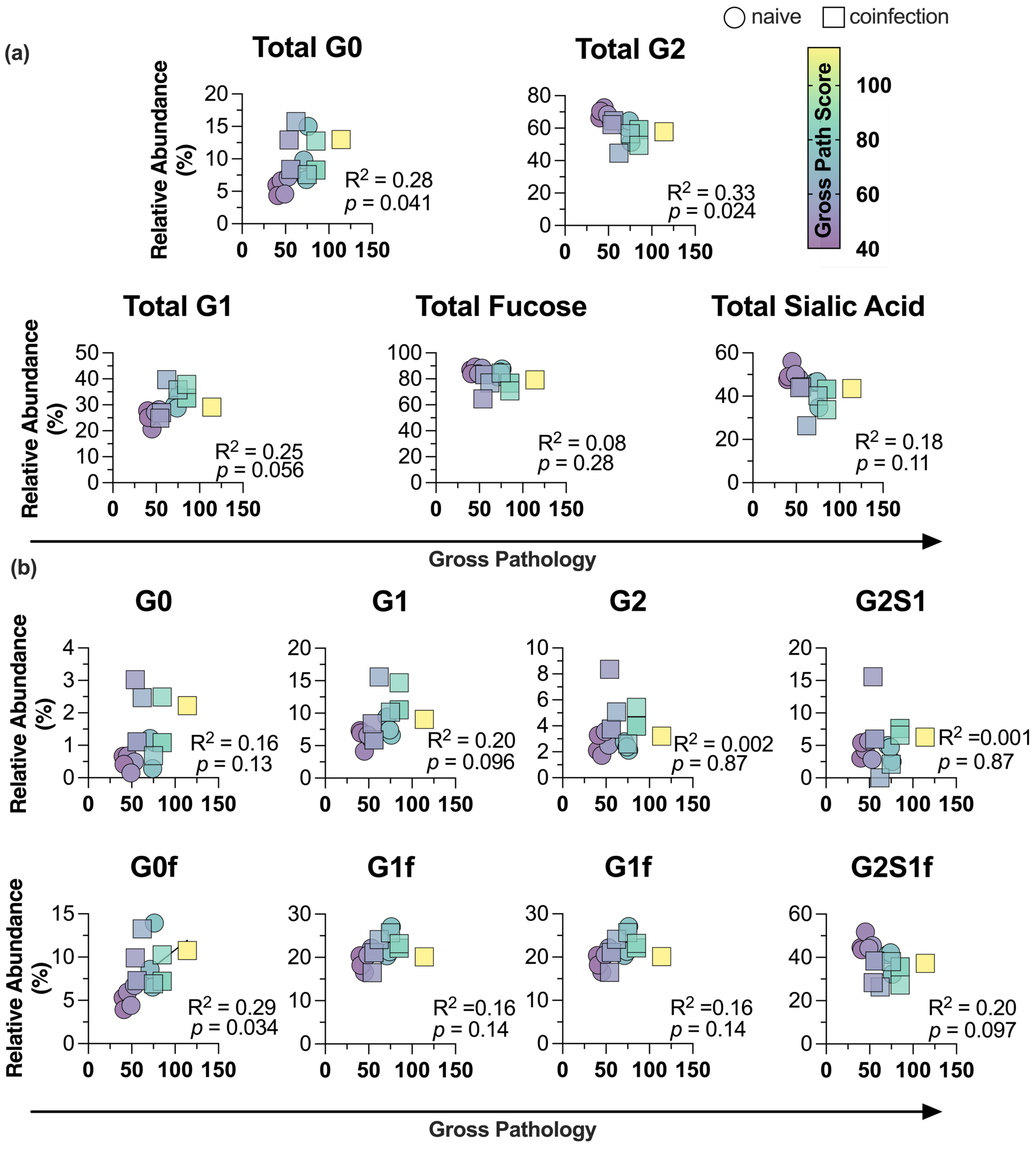

Supplementary Figure 6. Correlation of gross pathology scores with total and individual *N*-linked glycans eight weeks following *Mtb* infection. Linear correlation of the relative abundance of **(a)** total G0, total G1, total G2, total fucose, and total sialic acid and **(b)** individual glycans (G0, G0f, G1, G1f, G2, G2f, G2S1, G2S1f) eight weeks after *Mtb* infection with gross pathology scores for each animal. Simian immunodeficiency virus (SIV)-naïve animals (n = 8) are indicated by circles and SIV-coinfected animals (n =7) by squares, further coloured over a spectrum representing gross pathology score values (low score, dark purple; high score, yellow).
